# Reduced dose of PTCy followed by adjuvant α-galactosylceramide enhances GVL effect without sacrificing GVHD suppression

**DOI:** 10.1038/s41598-021-92526-z

**Published:** 2021-06-23

**Authors:** Makoto Nakamura, Yusuke Meguri, Shuntaro Ikegawa, Takumi Kondo, Yuichi Sumii, Takuya Fukumi, Miki Iwamoto, Yasuhisa Sando, Hiroyuki Sugiura, Noboru Asada, Daisuke Ennishi, Shuta Tomida, Emi Fukuda-Kawaguchi, Yasuyuki Ishii, Yoshinobu Maeda, Ken-ichi Matsuoka

**Affiliations:** 1 Department of Hematology and Oncology, Okayama University Graduate School of Medicine, Dentistry and Pharmaceutical Sciences, Okayama, Japan; 2 Department of Hematology and Oncology, Okayama University Hospital, Okayama, Japan; 3 Center for Comprehensive Genomic Medicine, Okayama University Hospital, Okayama, Japan; 4 REGiMMUNE Corporation, Tokyo, Japan; 5 Department of Urology, Tokyo Women’s Medical University, Tokyo, Japan; 6 Department of Immunological Diagnosis, Juntendo University Graduate School of Medicine, Tokyo, Japan

**Keywords:** Bone marrow transplantation, Allotransplantation

## Abstract

Posttransplantation cyclophosphamide (PTCy) has become a popular option for haploidentical hematopoietic stem cell transplantation (HSCT). However, personalized methods to adjust immune intensity after PTCy for each patient’s condition have not been well studied. Here, we investigated the effects of reducing the dose of PTCy followed by α-galactosylceramide (α-GC), a ligand of iNKT cells, on the reciprocal balance between graft-versus-host disease (GVHD) and the graft-versus-leukemia (GVL) effect. In a murine haploidentical HSCT model, insufficient GVHD prevention after reduced-dose PTCy was efficiently compensated for by multiple administrations of α-GC. The ligand treatment maintained the enhanced GVL effect after reduced-dose PTCy. Phenotypic analyses revealed that donor-derived B cells presented the ligand and induced preferential skewing to the NKT2 phenotype rather than the NKT1 phenotype, which was followed by the early recovery of all T cell subsets, especially CD4^+^Foxp3^+^ regulatory T cells. These studies indicate that α-GC administration soon after reduced-dose PTCy restores GVHD-preventing activity and maintains the GVL effect, which is enhanced by reducing the dose of PTCy. Our results provide important information for the development of a novel strategy to optimize PTCy-based transplantation, particularly in patients with a potential relapse risk.

## Introduction

Allogeneic hematopoietic stem cell transplantation (HSCT) is a curative therapy for patients with various hematological malignancies^1,2^. In recent years, HLA-haploidentical HSCT has helped overcome the limitations associated with the donor source, and posttransplant cyclophosphamide (PTCy) is an effective GVHD prophylaxis in this setting^3^. Studies exploring the mechanisms have shown that PTCy can reduce alloreactive T cells and induce functional impairment of surviving effector T cells while sparing regulatory T cells through the expression of aldehyde dehydrogenase^4–6^.

Although many clinical studies have shown that PTCy-based haploidentical HSCT has clinical superiority in terms of low non relapse-related mortality, concerns have been raised regarding the possible reduction in the graft-versus-leukemia (GVL) effect and the increased relapse rate of hematological cancer, especially in patients with an unfavorable disease status at transplantation or those requiring reduced-intensity conditioning^7–13^. In such cases, a full-dose of conditioning is often required to enhance the GVL effect and control tumors in patients, however, it may increase the risk of severe GVHD. Theoretically, PTCy dose reduction might generate GVL activity, however, reliable methods for recovering the weakened GVHD preventive activity after reduced-dose PTCy in patients receiving a full-dose of conditioning have not been well studied. Therefore, the development of an immunomodulatory strategy that can enhance the GVL effect while maintaining sufficient control of GVHD after PTCy-based HSCT is needed.

α-galactosylceramide (α-GC), a synthetic glycolipid originally isolated from the marine sponge *Agelas mauritianus*, is a representative ligand of invariant natural killer T (iNKT) cells^14^. α-GC is presented to iNKT cells by CD1d on antigen-presenting cells (APCs)^15,16^. Stimulated iNKT cells rapidly produce various cytokines, such as interferon (IFN)-γ, tumor necrosis factor (TNF), interleukin (IL)-4, IL-10 and IL-13^17–19^. In accordance with gene expression or cytokine profiling, it has been shown that iNKT cells express promyelocytic leukemia zinc finger (PLZF) and are generally divided into three phenotypes: type 1 helper T cell (Th1)-biased NKT (NKT1), NKT2, and NKT17, corresponding to Th1, Th2 and Th17 responses, respectively^20–24^.

Previous murine studies have shown that stimulation of iNKT cells by α-GC can induce immune tolerance in solid organ transplantation and HSCT^25–29^. In HSCT, α-GC stimulates host-type iNKT cells to produce large amounts of Th2 cytokines by a STAT6-dependent mechanism^27^ and promotes regulatory T cell (Treg) expansion in vivo^29^. Subsequently, a liposome formulation containing α-GC (lipo α-GC) was developed to efficiently and safely prevent acute GVHD and graft rejection by B cell-biased antigen presentation^26,28,30–33^. Based on these findings, a phase II clinical trial was performed and demonstrated that the administration of lipo α-GC immediately after HSCT contributed to the prevention of acute GVHD and that this effect was dependent on Treg expansion^34^.

In this study, we first examined whether PTCy dose reduction can enhance the GVL effect. We then examined whether lipo α-GC as an immune adjuvant can compensate for the reciprocal balance between the GVL effect and GVHD prevention after modulating the PTCy dose. This study aimed to demonstrate the possibility of a novel immune-modulatory strategy to optimize PTCy-based transplantation, particularly for patients with a potential relapse risk.

## Results

### PTCy ameliorates GVHD in a dose-dependent manner

**Figure 1:**
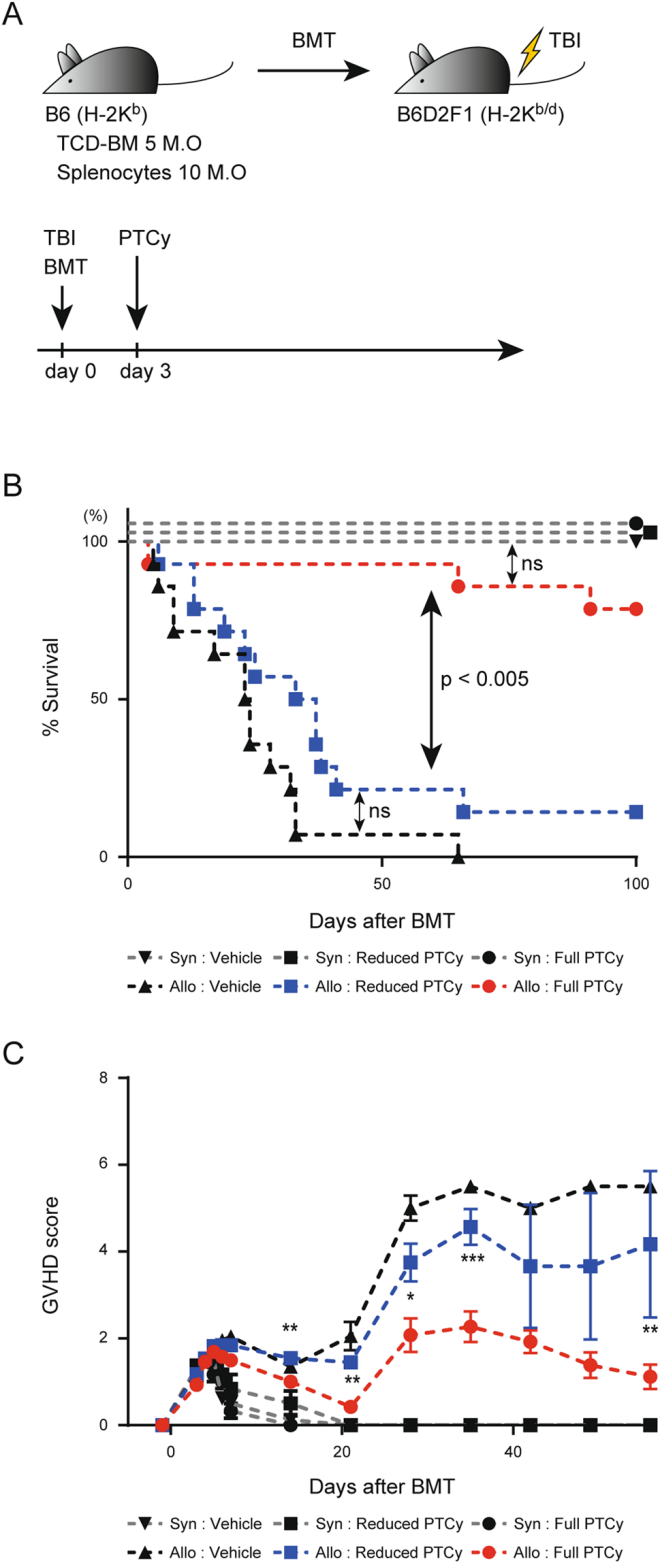
GVHD is prevented by optimized treatment with PTCy. (**A**) Experimental scheme: 5 × 10^6^ TCD-BM cells and 10 × 10^6^ splenocytes from B6 donor mice were administered to lethally irradiated (12 Gy TBI) B6D2F1 recipient mice. Cyclophosphamide or a control vehicle was administered on day 3 after BMT. (**B**) Overall survival rates of the vehicle (n = 14), reduced PTCy (n = 14) and full PTCy (n = 14) groups at serial time points. Data are representative of four independent experiments. *P* values were determined by the log-rank test and Holm’s adjustment for multiple comparisons. ns, not significant (*P* > 0.05). (**C**) Clinical scores of the vehicle (n = 14), reduced PTCy (25 mg/kg) (n = 14) and full PTCy (50 mg/kg) (n = 14) groups at serial time points. Data are representative of four independent experiments and expressed as the mean ± SEM. *P* values were determined by one-way ANOVA and Tukey’s adjustment for multiple comparisons (allogeneic: reduced PTCy vs allogeneic: full PTCy). *P < 0.05; **P < 0.01; ****P < 0.0001.

We first assessed the GVHD-preventing impact of PTCy on a haploidentical BMT model. Transplantation of 5 × 10^6^ T cell-depleted bone marrow (TCD-BM) cells and 10 × 10^6^ splenocytes from donor C57BL/6 J (B6) mice into lethally irradiated recipient B6D2F1 mice was carried out, followed by PTCy administration 3 days after BMT (Fig. 1A). We used 12 Gy of the total body irradiation (TBI) as a lethal irradiation in this experimental setting. A pilot experiment to identify the optimal dose of cyclophosphamide for PTCy showed that PTCy at a dose of 50 mg/kg significantly protected recipients from acute GVHD, while a dose of 100 mg/kg failed to do so (Fig. S1). Therefore, we decided to use 50 mg/kg cyclophosphamide, defined as “Full PTCy”, to prevent GVHD effectively in our murine BMT model. Next, the impact of a half dose of cyclophosphamide, defined as “Reduced PTCy”, was tested in the same model. The overall survival rate of the transplanted cohorts was improved by full PTCy (red closed circle) but not the reduced PTCy (blue closed square) as compared to allogeneic control (black closed triangle) (Fig. 1B). The clinical GVHD score of the full PTCy cohort (red closed circle) was significantly lower than that of the reduced PTCy cohort (blue closed square) on days 14, 21, 28, 35 and 56 (Fig. 1C). These results indicate that the GVHD-preventing effect is dependent on the PTCy-dose, and a reduced PTCy of 25 mg/kg in this model was not sufficient to control GVHD compared to a full PTCy of 50 mg/kg.

### Additional administration of lipo α-GC could compensate for the insufficient GVHD prevention after reduced-dose PTCy

**Figure 2:**
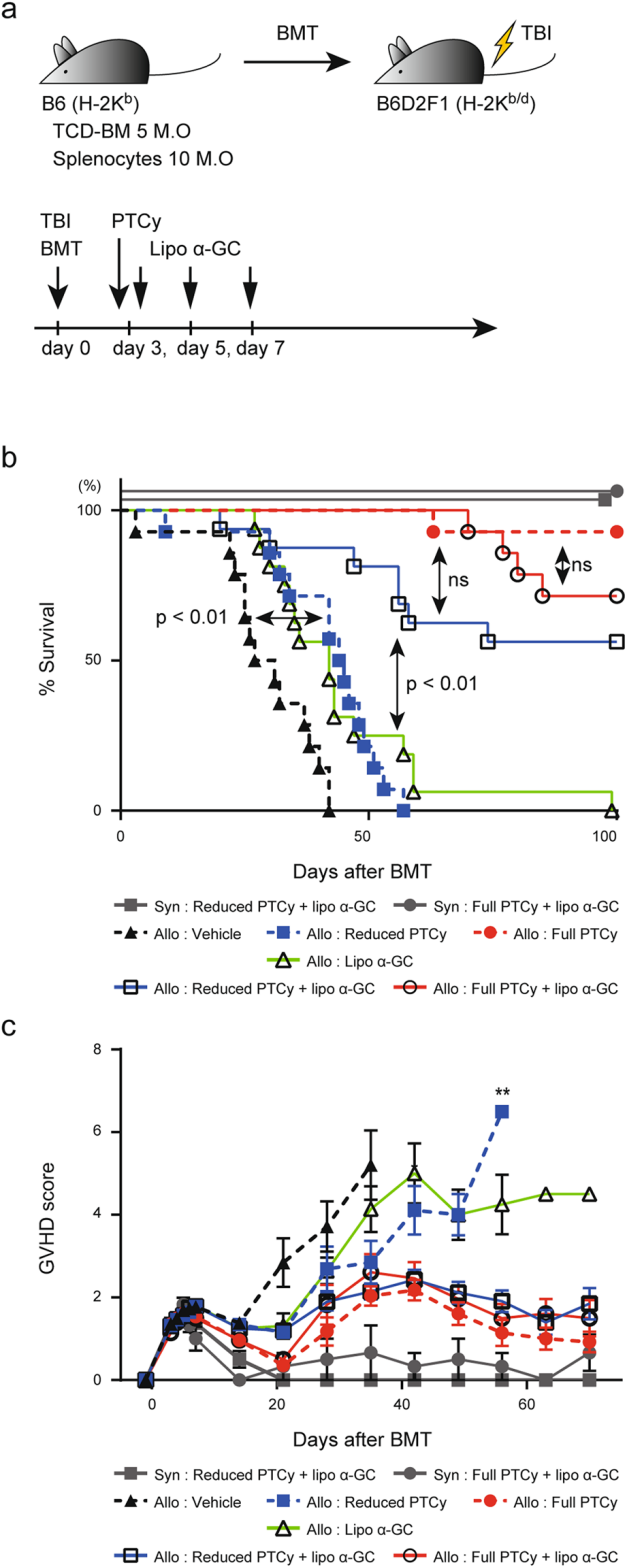
Lipo α-GC improves both the percent survival and the GVHD score impaired by reducing the dose of PTCy. (**a**) Experimental scheme: 5 × 10^6^ TCD-BM cells and 10 × 10^6^ splenocytes from B6 donor mice were administered to lethally irradiated (12 Gy TBI) B6D2F1 recipient mice. Cyclophosphamide or a control vehicle was administered on day 3, followed by lipo α-GC administration on days 3, 5 and 7 after BMT. (**b**) Overall survival rates of the vehicle (n = 14), reduced PTCy (n = 14), full PTCy (n = 14), lipo α-GC (n = 16), reduced PTCy plus lipo α-GC (n = 16) and full PTCy plus lipo α-GC (n = 14) groups at serial time points. Data are representative of four independent experiments. *P* values were determined by the log-rank test and Holm’s adjustment for multiple comparisons. ns, not significant (*P* > 0.05). (**c**) Clinical scores of the vehicle (n = 14), reduced PTCy (n = 14), full PTCy (n = 14), lipo α-GC (n = 16), reduced PTCy plus lipo α-GC (n = 16) and full PTCy plus lipo α-GC (n = 14) groups at serial time points. Data are representative of four independent experiments and expressed as the mean ± SEM. *P* values were determined by one-way ANOVA and Tukey’s adjustment for multiple comparisons (allogeneic: reduced PTCy vs allogeneic: reduced PTCy plus lipo α-GC). *P < 0.05.

To examine the effect of lipo α-GC on GVHD preventive activity in our experiment, we attempted a lipo α-GC treatment with or without PTCy after transplantation (Fig. 2a). Although each lipo α-GC monotherapy (black open triangle) or reduced PTCy monotherapy (blue closed square) resulted in prolonged survival as compared to allogeneic control cohort without receiving any therapies (violet closed triangle), the effects were just limited (Fig. 2b). Interestingly, the combination therapy of reduced PTCy and lipo α-GC (black open square) remarkably improved the overall survival and GVHD clinical scores as compared to the monotherapy with reduced PTCy (blue closed square), suggesting that the reduced PTCy and lipo α-GC synergistically prevented GVHD (Fig. 2b,c). These results indicated that the additional administration of lipo α-GC could compensate for the insufficient GVHD prevention mediated by reduced-dose PTCy alone.

### Full PTCy reduced the GVL effect, while administration of lipo α-GC did not

Since post-transplant relapse is one of the leading causes of death after transplantation as well as GVHD, it is critical that sufficient GVL effect is ensured after the administration of each PTCy or lipo α-GC which may deeply affect donor effector T cell function. We therefore conducted the experiments to investigate whether the GVHD-preventing activity by PTCy or by lipo αGC could lead to the reduction of the GVL effect or not.

**Figure 3:**
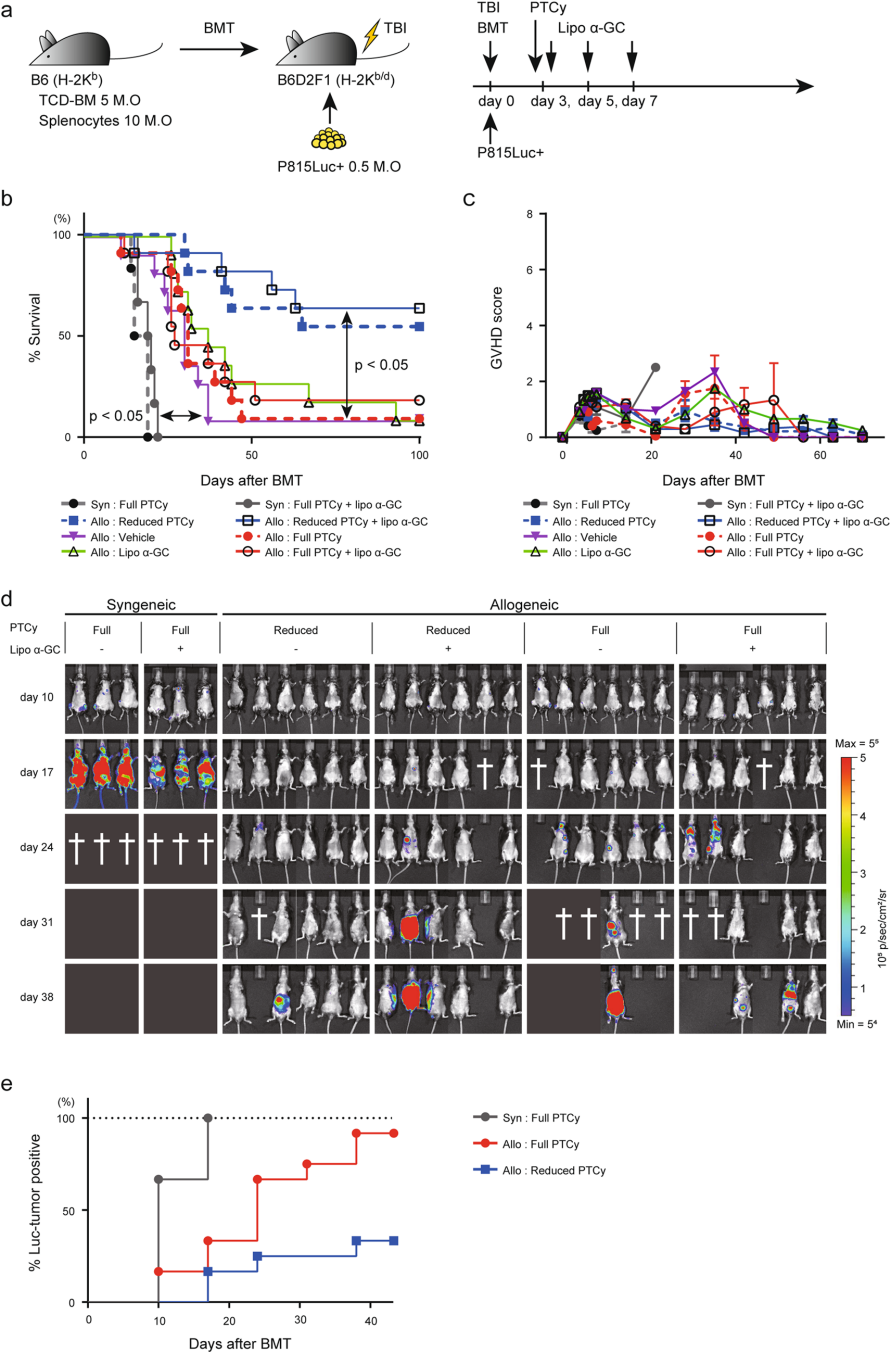
Full PTCy reduced the GVL effect, while administration of lipo α-GC did not. (**a**) Experimental scheme: 5 × 10^6^ TCD-BM cells and 10 × 10^6^ splenocytes from B6 donor mice were administered to sublethally irradiated (8 Gy TBI) B6D2F1 recipient mice, and 5 × 10^5^ luciferase-expressing P815 cells were also administered. Cyclophosphamide or a control vehicle was administered on day 3, followed by lipo α-GC administration on days 3, 5, and 7 after BMT. (**b**,**c**) Overall survival rates and clinical scores of the syngeneic: full PTCy (n = 6) or full PTCy plus lipo α-GC (n = 6) and allogeneic: vehicle (n = 11), lipo α-GC (n = 11), reduced PTCy (n = 11), full PTCy (n = 11), reduced PTCy plus lipo α-GC (n = 11) or full PTCy plus lipo α-GC (n = 11) groups at serial time points. Data are representative of two independent experiments. *P* values were determined by the log-rank test and Holm’s adjustment for multiple comparisons. (**d**) IVIS study of the syngeneic: full PTCy (n = 3) or full PTCy plus lipo α-GC (n = 3) and allogeneic: reduced PTCy (n = 6), full PTCy (n = 6), reduced PTCy plus lipo α-GC (n = 6) or full PTCy plus lipo α-GC (n = 6) groups at serial time points. (**e**) The percentage of positive tumor signal over time in syngeneic group (n = 6), allogeneic with full PTCy (n = 12) and allogeneic with reduced PTCy (n = 12). In each group, the cohorts with and without lipo α-GC were combined.

To evaluate the GVL activity, we set up tumor-bearing recipients by transferring luciferase-expressing P815 cells (Fig. 3a). To minimize the relative impact of PTCy on the tumor and evaluate the GVL effect of the donor’s immunity, the tumor volume was carefully titrated and set to the maximum amount that does not cause acute death. As for dose of RT, we attempted to examine the GVL activity under the condition with TBI 12 Gy and 10 Gy, as near settings as in Fig. 2; however, recipients after reduced-PTCy died within 50 days after transplant by GVHD and it was impossible to evaluate the GVL activity. We therefore used a lower TBI dose (8 Gy) which enabled to reduce the GVHD intensity and assess the tumor-related lethality over 100 days.

In the syngeneic settings, the survival rates were decreased in proportion to the onset of tumor-specific clinical features such as leg paralysis or macroscopic tumor infiltration into the liver and spleen at approximately 3 or 4 weeks after BMT (black and gray closed circles) (Fig. 3b). Allogeneic cohorts without PTCy (violet closed triangle) or with full PTCy (red closed circle) significantly prolonged the survival compared to syngeneic cohorts (black and gray closed circles), however, a major proportion of recipients died until 12 weeks. In contrast, allogeneic cohort with reduced PTCy (blue closed square) remarkably improved overall survival more than full PTCy (red closed circle). The addition of lipo α-GC treatment after reduced or full PTCy (black open square and black open circle, respectively) resulted in the comparable survival as compared to the corresponding cohorts receiving PTCy alone (blue closed square and red closed circle, respectively) (Fig. 3b). The clinical GVHD scores in all eight cohorts were maintained at low levels and did not reach fatal GVHD severity throughout the experimental period (Fig. 3c). Also, there was no difference in GVHD severity among the 8 cohorts. This data suggest that the main cause of death in this experimental setting was tumor progression rather than GVHD. Quantitative analysis using an in vivo imaging system (IVIS) enabled monitoring of the progression of the inoculated luciferase-expressing P815 cells in each mouse. As early as day 10, tumor signaling was detected in some recipients in the cohorts without PTCy, but not in the cohorts with PTCy, suggesting that PTCy itself has a certain impact on tumor control (Fig. 3d,S2). The percentage of positive tumor signal over time was clearly suppressed in the “Reduced PTCy” cohort (blue closed square) compared to the “Full PTCy” cohort (red closed circle) (Fig. 3d,e).

These results suggested that full PTCy could reduce the GVL effect resulting in the tumor-related mortality in recipients with a high tumor burden. In contrast, the additional administration of lipo α-GC appeared not to affect the GVL activity.

### Lipo α-GC administered after PTCy was delivered to B cells and macrophages rather than dendritic cells

**Figure 4:**
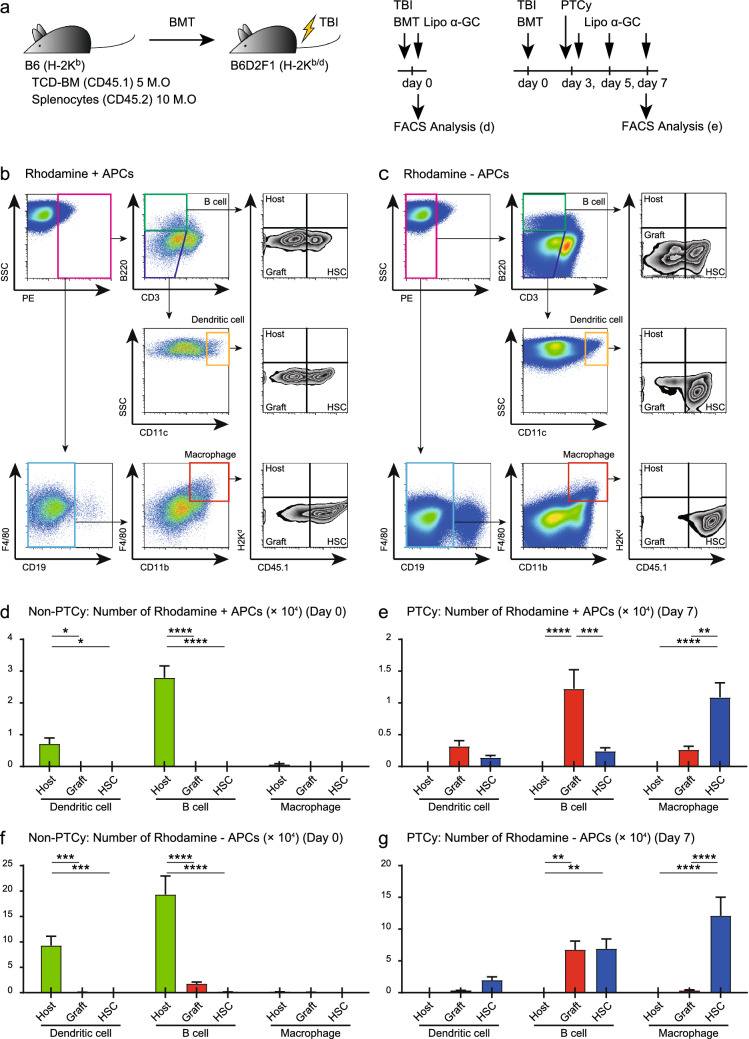
APCs presenting lipo α-GC vary by phase after BMT. (**a**) 5 × 10^6^ TCD-BM cells from B6 (H-2KbCD45.2 +) donor mice and 5 × 10^6^ splenocytes from Ly5.1-B6 (H-2KbCD45.1 +) donor mice were administered into lethally irradiated B6D2F1 (H-2 Kb/dCD45.2 +) recipient mice. In non-PTCy group, rhodamine-labeled lipo α-GC was administered within 30 min after transplantation. Two hours after the administration, splenocytes were collected from donor. In PTCy group, reduced dose of 25 mg/kg cyclophosphamide was administered intraperitoneally into recipient mice on day3. Lipo α-GC was administered intravenously on day 3, 5. On day 7, rhodamine-labeled lipo α-GC was administered. Two hours after the administration, splenocytes were collected from donor. Rhodamine uptake by APCs was assessed using flow cytometric analysis. (**b**,**c**) Representative data of flow cytometric analysis regarding Rhodamine positive or negative APCs are shown. Each gated cells were divided into B cells (CD3-B220 +), dendritic cells (CD3-CD11chigh) and macrophages (CD19-F4/80highCD11bhigh). And, their chimeras are evaluated as host (H2Kd + CD45.1-) derived or donor graft (H2Kd-CD45.1-) derived or donor stem cell (H2Kd-CD45.1 +) derived, respectively. (**d**) In non-PTCy group, APCs taking in lipo α-GC and their chimeras on day 0 immediately after BMT are revealed by flow cytometric analysis (n = 6). (**e**) In PTCy group, APCs taking in lipo α-GC and their chimeras on day7 after BMT are revealed, by flow cytometric analysis (n = 6). (**f**) In non-PTCy group, APCs not taking in lipo α-GC and their chimeras on day 0 immediately after BMT are revealed by flow cytometric analysis (n = 6). (**g**) In PTCy group, APCs not taking in lipo α-GC and their chimeras on day7 after BMT are revealed, by flow cytometric analysis (n = 6). Data are expressed as means ± SEM. *P* values by one-way ANOVA and the Tukey’s adjustment for multiple comparison. *P < .05; **P < .01; ***P < .001; ****P < .0001.

To identify which APC incorporated lipo α-GC administered after PTCy, we used a rhodamine-labeled lipo α-GC. In our previous report, lipo α-GC preferentially accumulated in splenic marginal zone B cells in normal mice within 2 h after intravenous injection^32^. We confirmed whether the target cells of lipo α-GC in normal mice were the same as those in the murine haploidentical BMT model. Lethally irradiated mice were intravenously injected with rhodamine-labeled lipo α-GC immediately after BMT, and their spleens were extracted 2 h after the injection (middle, in Fig. 4a). Splenic B cells, dendritic cells (DCs) or macrophages incorporating lipo α-GC were defined as B220^+^CD3^−^, B220^−^CD3^−^CD11c^+^ or CD19^−^F4/80^+^CD11b^+^ cells in the rhodamine-positive gate of flow cytometric analysis, respectively (Fig. 4b). In contrast, cells not incorporating lipo α-GC were defined by the same gating in the rhodamine-negative gate (Fig. 4c).

In the non-PTCy conventional BMT model, rhodamine-positive cells mostly corresponded to host-derived B cells rather than DCs, but not to the donor graft-derived APC (Fig. 4d). Conversely, in the case of lipo α-GC treatment after PTCy, rhodamine-positive cells were mainly composed of graft-derived B cells and host stem cell (HSC)-derived macrophages (Fig. 4e). These results suggest that lipo α-GC is present in graft-derived B cells and stem cell-derived macrophages rather than DCs in the PTCy-based haploidentical transplant setting. We also checked the composition of APCs in the rhodamine-negative cells in non-PTCy conventional (Fig. 4f) and PTCy (Fig. 4g) BMT models, respectively. The comparison between Fig. 4e,g indicates that the percentage of the graft-derived subset in rhodamine-positive cells is relatively higher than that in rhodamine-negative cells.

### Splenic T cells have a good potential to deliver lipo α-GC after BMT

**Figure 5:**
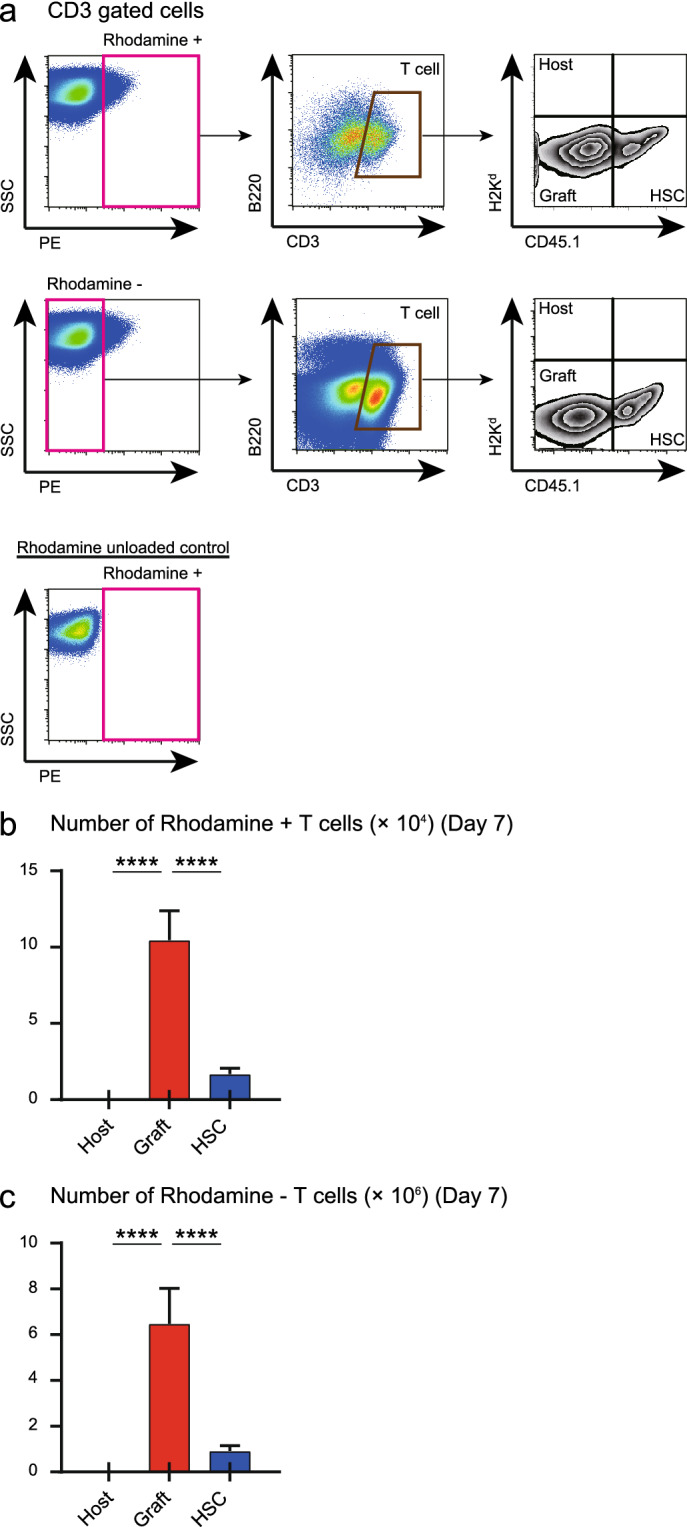
Splenic T cells uptake lipo α-GC after BMT. Splenic T cells were analyzed on day 7 after murine BMT with reduced PTCy as shown in Fig. 4a. (**a**) Representative data of flow cytometric analysis regarding Rhodamine positive or negative CD3 + T cells are shown. Their chimeras are evaluated as host (H2Kd + CD45.1-) derived or donor graft (H2Kd-CD45.1-) derived or donor stem cell (H2Kd-CD45.1 +) derived, respectively. (**b**) APCs taking in lipo α-GC and their chimeras on day7 after BMT are revealed, by flow cytometric analysis (n = 6). (**c**) APCs not taking in lipo α-GC and their chimeras on day7 after BMT are revealed, by flow cytometric analysis (n = 6).

In addition to B cells, macrophages, and DCs, CD1d is universally expressed in various cells and tissues. Therefore, we examined whether splenic T cells incorporated lipo α-GC after PTCy. Splenic T cells were analyzed on day 7 after murine BMT with reduced PTCy as shown in Fig. 4a. Rhodamine positive fraction involved CD3^+^ cells, suggesting that some population of CD3^+^ T cells can uptake lipo α-GC (Fig. 5a). A major part of Rhodamine positive T cells was donor graft-derived, and the absolute number was greater than that of Rhodamine positive APCs. This suggests that both B cells and T cells play an important role in delivering lipo α-GC after BMT.

### Profile of iNKT cell subsets skewed toward the Th2 phenotype after PTCy with lipo α-GC treatment

**Figure 6:**
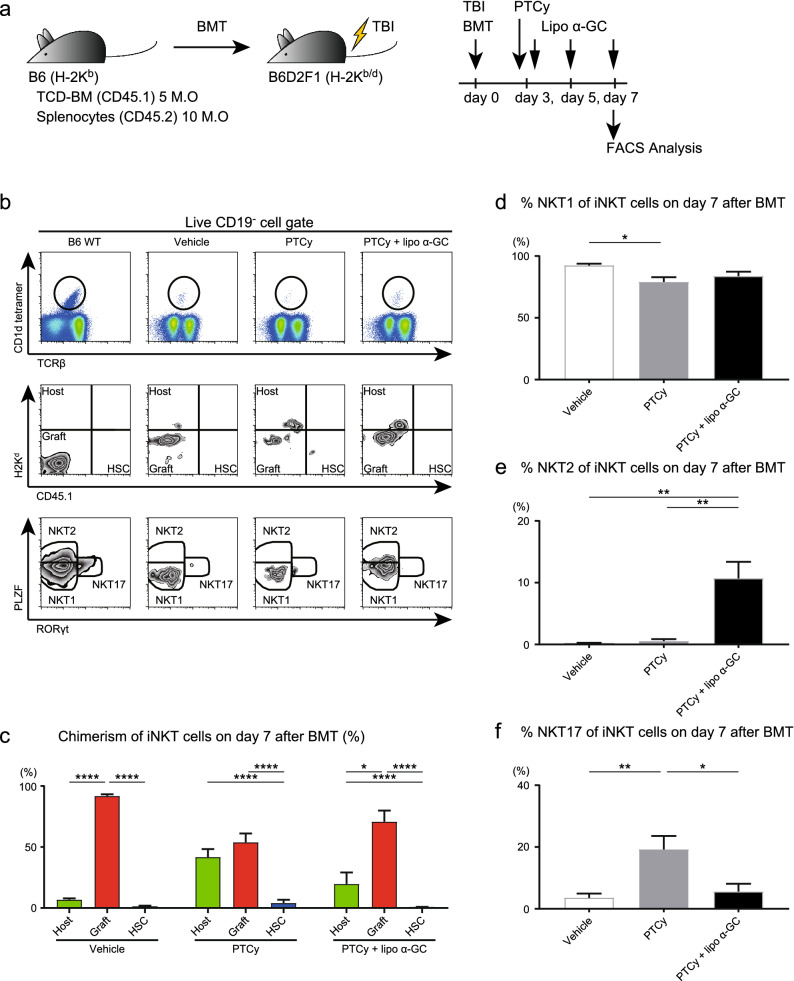
Th2-phenotype iNKT cells are expanded by “Reduced PTCy” with lipo α-GC. (**a**) Experimental scheme: 5 × 10^6^ TCD-BM cells from B6 (H-2K^b^CD45.2^+^) donor mice and 10 × 10^6^ splenocytes from Ly5.1-B6 (H-2K^b^CD45.1^+^) donor mice were administered to lethally irradiated B6D2F1 (H-2 Kb^/d^CD45.2^+^) recipient mice. A reduced dose of 25 mg/kg cyclophosphamide or control vehicle was administered on day 3, followed by lipo α-GC administration on days 3, 5, and 7 after BMT. On day 7 after BMT, the phenotypes of iNKT cells (live, CD19^−^α-GC-loaded CD1d tetramer^+^TCRβ^+^) were analyzed as follows: NKT1 (PLZF^low^RORγt^−^), NKT2 (PLZF^high^RORγt^−^), and NKT17 (PLZF^int^RORγt^−^). (**b**) Representative data from a flow cytometric analysis showing iNKT cells, their phenotypes and chimeras in the vehicle (n = 5), reduced PTCy (n = 5) and lipo α-GC plus reduced PTCy (n = 5) groups. (**c**) Chimerism of the iNKT cells in the vehicle (n = 5), reduced PTCy (n = 5) and lipo α-GC plus reduced PTCy (n = 5) groups. (d,e,f) Percentage of cells with each phenotype among the iNKT cells in the vehicle (n = 5), reduced PTCy (n = 5) and lipo α-GC plus reduced PTCy (n = 5) groups. *P* values were determined by one-way ANOVA and Tukey’s adjustment for multiple comparisons. *P < 0.05; **P < 0.01; ***P < 0.001; ****P < 0.0001.

We wondered whether the unique profile of the APCs incorporating lipo α-GC influences the proportion of each phenotype of iNKT cells. Splenocytes were prepared immediately after the third injection of lipo α-GC into the model mice (Fig. 6a). NKT1, NKT2 and NKT17 cells were defined as PLZF^low^RORγt^−^, PLZF^high^RORγt^−^ and PLZF^int^RORγt^+^ cells, respectively, in the iNKT cell population (Fig. 6b). The analysis of the chimerism of splenic iNKT cells showed that the donor/host cell ratio was significantly higher in the mice treated with PTCy and lipo α-GC, while the ratio in those receiving PTCy monotherapy was mostly equal (Fig. 6c). Additionally, a flow cytometric analysis showed that the proportion of cells with the NKT2 phenotype was remarkably increased after PTCy with lipo α-GC treatment, but not after PTCy monotherapy or non-treatment; however, the proportion of cells in the NKT1 subset was comparable among all treatments (Fig. 6d,e). The proportion of NKT17 cells after PTCy monotherapy was significantly higher than those following other treatments, although the absolute number remains very low (Fig. 6f), suggesting that the expansion of NKT2-phenotype cells by PTCy with lipo α-GC treatment could inhibit the expansion of NKT17-phenotype cells induced by PTCy alone.

### Lipo α-GC enhances the expansion of donor graft-derived Tregs

**Figure 7:**
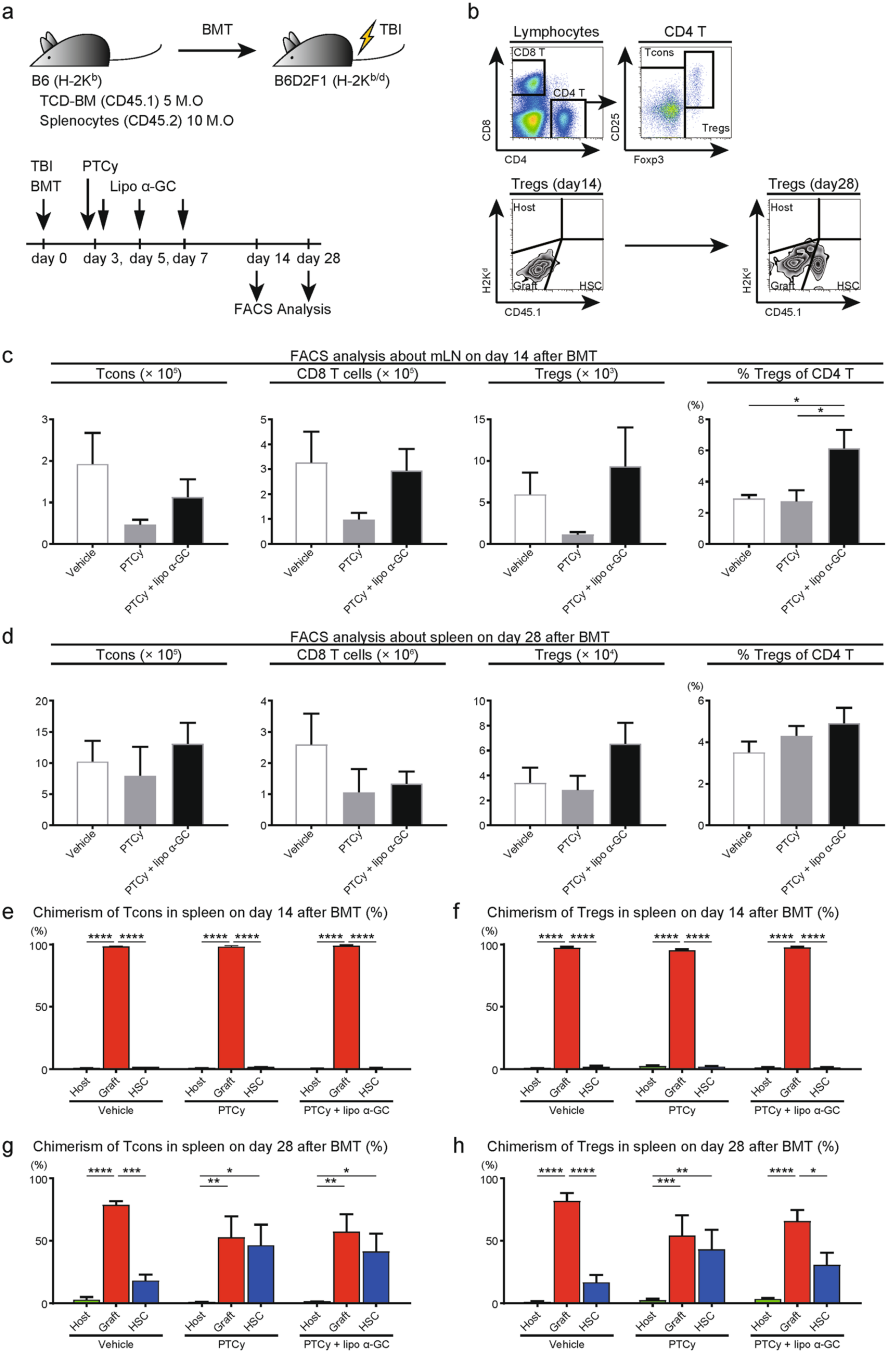
Lipo α-GC enhances the expansion of donor-derived Tregs. (**a**) Experimental scheme: 5 × 10^6^ TCD-BM cells from B6 (H-2K^b^CD45.2^+^) donor mice and 10 × 10^6^ splenocytes from Ly5.1-B6 (H-2K^b^CD45.1^+^) donor mice were administered to lethally irradiated B6D2F1 (H-2 Kb^/d^, CD45.2^+^) recipient mice. A reduced dose of 25 mg/kg cyclophosphamide or control vehicle was administered on day 3, followed by lipo α-GC administration on days 3, 5, and 7 after BMT. (**b**) Representative data from a flow cytometric analysis show Tregs (CD4^+^Foxp3^+^CD25^+^), Tcons (CD4^+^Foxp3^−^) and CD8^+^ T cells in the splenocyte population and the associated chimerism after BMT in the vehicle (n = 7), reduced PTCy (n = 8) and lipo α-GC plus reduced PTCy (n = 7) groups. (**c**) Flow cytometric analysis of mLN cells on day 14 after BMT revealed the absolute numbers of Tcons, CD8^+^ T cells and Tregs and percentages of Tregs. (**d**) Flow cytometric analysis of splenocytes on day 28 after BMT revealed the absolute numbers of Tcons, CD8^+^ T cells and Tregs and percentages of Tregs. (**e**,**f**) Flow cytometric analysis of splenocytes on day 14 after BMT revealed the chimerism of Tcons and Tregs. (**g**,**h**) Flow cytometric analysis of splenocytes on day 28 after BMT revealed the chimerism of Tcons and Tregs. *P* values were determined by one-way ANOVA and Tukey’s adjustment for multiple comparisons. *P < 0.05; **P < 0.01; ***P < 0.001; ****P < 0.0001.

We then examined the impact of lipo α-GC on Tregs in our PTCy-based transplantation model (Fig. 7a). Each subset of T cells and their origin were defined as shown in the upper and lower panels of Fig. 7b, respectively. On day 14 after BMT, flow cytometric analysis of mesenteric lymph node (mLN) cells and splenocytes showed that PTCy with lipo α-GC induced significantly higher percentages of Tregs (*P* < 0.05) than the control vehicle or PTCy alone (Fig. 7c,S3a). The combined PTCy and lipo α-GC also increased CD8^+^ T cells, but the ratio of Treg and CD8^+^ T cells in this treatment group was higher than that in the other groups, suggesting that Treg increased predominantly and contributed to immune stability (Fig. S4).

On day 14, the majority of Tregs were derived from the donor graft (Fig. 7f). Analysis on day 28 showed that PTCy appeared to promote the emergence of donor stem cell-derived T cells including Tregs (Fig. 7g,h), and Treg levels in PTCy with lipo α-GC were maintained as compared with other groups (Fig. 7d,S3b). These results indicated that PTCy with lipo α-GC treatment could induce the expansion of donor graft-derived Tregs early after BMT, consequentially followed by the early emergence of donor stem cell-derived Tregs.

## Discussion

It is clinically desirable that both GVHD prevention and GVL activation are achieved after HSCT, particularly in the setting of PTCy-based haploidentical transplantation in patients with a potential relapse risk. Although PTCy dose reduction might be assumed to generate GVL activity, it is not applicable in human clinical treatment due to unavailable reliable methods for recovery of the GVHD-preventing activity weakened by reducing the dose of PTCy. In our murine haploidentical transplantation model, we showed that lipo-α-GC could induce the regulatory functions of iNKT cells and perform a complementary role in preventing GVHD, combined with a reduced-dose PTCy. We also demonstrated that lipo α-GC could maintain a powerful GVL effect generated by reduced-dose PTCy.

We optimized a murine model of PTCy-based transplantation in a haploidentical setting to properly evaluate the effect of lipo α-GC on PTCy. In this transplant setting, 50 mg/kg of PTCy was sufficient to prevent acute GVHD (Fig. 1B,C). PTCy at a dose of 100 mg/kg resulted in worse survival than 50 mg/kg of PTCy, suggesting that this dose might induce lethal side effects, such as cardiomyopathy (Fig. S1). We defined 50 mg/kg of PTCy as full PTCy as this was a safe dose that avoids drug-induced lethal side effects and effective in suppressing acute GVHD.

A previous murine study examining the biological effects of two doses of PTCy (22 mg/kg and 66 mg/kg) demonstrated that PTCy diminished alloreactive proliferating T cell numbers in a dose-dependent manner^35^. In our study, a reduced PTCy, defined as a dose of 25 mg/kg, has insufficient GVHD prevention, and most recipients failed to survive to day 50. Meanwhile, a full PTCy efficiently prevented GVHD and resulted in survival rates comparable to syngeneic controls (Fig. 1B,C). These results indicate that our optimized BMT model is suitable for monitoring PTCy dose-dependent effects on GVHD prevention and overall survival prolongation.

The impact of α-GC on GVHD suppression by iNKT-mediated Treg expansion has been reported in murine models and clinical studies^25,26,34^. Similar to these studies, our current study also showed that lipo α-GC monotherapy prolonged overall survival (Fig. 2a). However, the effect of α-GC has not been evaluated in a PTCy-based HSCT setting. Our data showed that lipo α-GC synergistically operated to recover the lost preventive activity against GVHD caused by reducing the PTCy dosage (Fig. 2b,c).

We then evaluated the GVL effect after PTCy using luciferase-expressing P815 tumor cells with a modified TBI (Fig. 2,3). Results showed that both full and reduced PTCy prevented tumor signals at day 10, but thereafter, significantly prolonged survival was observed in the reduced PTCy cohort than the full PTCy cohort. This indicates that although high-dose PTCy can provide tumor control immediately after transplantation, GVL attenuation by PTCy can have a substantial impact, and reducing the dose of PTCy can strengthen the GVL effect.

Notably, GVL after PTCy was not affected by lipo α-GC treatment (Fig. 3b). This is consistent with a previous study using a non-PTCy BMT model, which demonstrated that a single injection of lipo α-GC immediately after BMT in a P815 cell-inoculated murine acute GVHD model resulted in prolonged survival in recipient mice without loss of GVL activity^26^.

As shown above, we set two different experimental settings in this study; one is to assess GVHD (TBI 12 Gy; Fig. 1,2), and the other is to assess GVL effect (TBI 8 Gy; Fig. 3,S2). Since the assessment of the long-term GVL effect was not possible due to the early GVHD-related mortality in the setting of lethal TBI (12 Gy), we used another setting with sublethal TBI (8 Gy) to evaluate the GVL effect. In this setting, GVHD was mild, and recipients could survive as long as the tumor was controlled by the GVL activity (Fig. 3c). It is difficult to accurately evaluate the GVL effect in the current murine HSCT model with lethal TBI as GVHD-related death can occur. This is an important limitation in translating basic findings to clinical situations and should be resolved in the future.

Our data demonstrated that lipo α-GC did not reduce the GVL activity in the setting with sublethal TBI (Fig. 3). It is true that the effect of lipo α-GC on GVL in the setting of lethal TBI was not evaluated in this study, however, we consider that it would be just limited because the GVL activity should be more enhanced after lethal TBI as compared to sublethal TBI. A previous murine study demonstrated that higher TBI is better to control P815 tumors when compared to that with reduced TBI by using the same system as our current study (B6 into B6D2F1)^36^. Consistent with this basic experiment, many clinical studies have already clarified that a higher dose of conditioning contributes to control tumors as compared to a lower dose^37,38^. These previous findings suggest that the negative effect of lipo α-GC on the GVL activity could be further diminished or offset in the setting of lethal TBI. This is a very important point and should be carefully verified in future clinical trials.

To detect the main cells that present lipo α-GC, a rhodamine uptake assay was performed. Analysis following a single injection of lipo α-GC after BMT without PTCy showed that the splenic target cells incorporating lipo α-GC were mostly B cells derived from recipient mice. Meanwhile, the analysis following multiple injections after PTCy found that the target cells were B cells derived from the donor graft and macrophages from the reconstituted HSC population (Fig. 4d,e). Sonoda et al. reported that splenic marginal zone B cells promoted NKT cell-dependent tolerance^39^. In contrast, Arora et al. reported that various glycolipid antigens were presented mainly by DCs, not by B cells, and whether iNKT cells develop Th1- or Th2-response by lipo α-GC was determined by changes in the surface markers of DCs, consisting of various costimulatory and coinhibitory molecules^40^. Bezbradica et al. showed that B cells presenting α-GC preferentially induced IL-4 rather than IFN-γ production by NKT cells^41^. Bai et al. demonstrated that compared with DCs, B cells stimulated iNKT cells more effectively when loaded with Th2-biased ligands than when loaded with conventional α-GC and induced relatively more IL-4 than IFN-γ expression^42^. In our previous study, we administered lipo α-GC to mice and sorted the B cells. The sorted B cells were co-cultured with iNKT cells in vitro, and IL-10 production was induced without inducing IFN-γ production^32^. Results from the in vitro experiment suggested that iNKT cells can be promoted into NKT2-shift by stimulating with B cells that incorporated lipo α-GC. In a posttransplant environment, we believe that DCs are easily stimulated and activated by inflammatory cytokines and mediate Th1 skewing of NKT cells; therefore, B cell acquisition and presentation of α-GC is theoretically advantageous with respect to Th2 skewing of NKT cells.

Interestingly, some CD3^+^ T cell populations also showed rhodamine-positive cells (Fig. 5a,b). Previous reports have demonstrated that CD1d is also expressed in T cells^43^. Furthermore, a recent study analyzed the function of CD1d expressed on T cells and concluded that CD1d-expressing CD8^+^ T cells present antigens to iNKT cells, producing IFN-γ, resulting in an enhanced antitumor effect^44^. In our data, α-GC administration tended to prolong survival in the cohorts without PTCy (Fig. 3,S2), suggesting that donor-derived CTLs directly incorporating α-GC might exert an anti-tumor effect.

The function of the macrophages incorporating lipo α-GC remains unclear. It has been shown that macrophages promote IL-4 production by iNKT cells when cultured in vitro with Th2-biased ligands^42^. Other studies have reported that macrophages play critical roles in the distribution and migration of iNKT cells in vivo^45,46^. Thus, the HSC-derived macrophages incorporating lipo α-GC may have had some impact on iNKT homeostasis in our transplantation experiments. However, further studies are required to clarify the detailed functions of macrophages.

Flow cytometry analysis further subdivided NKT cells into NKT1, NKT2, and NKT17 cells according to the expression pattern of PLZF and RORγt (Fig. 6). Results showed that iNKT cells skewed toward the Th2 phenotype by PTCy with lipo α-GC treatment, indicating the impact of lipo α-GC on the phenotypic plasticity of iNKT cells in the posttransplant peripheral inflammatory environment (Fig. 6b). We previously demonstrated that host-derived iNKT cells and IL-4 production are required for the suppression of GVHD by α-GC^27,29,47^. In other studies, adoptive transfer of donor-type iNKT cells ameliorated GVHD via donor-derived Treg expansion^48,49^. Moreover, it has been demonstrated that even third-party iNKT cells prevent GVHD via early expansion of Tregs, even though these iNKT cells are rejected shortly after administration^50^. Our data on NKT2 skewing are in line with the data demonstrating B cell acquisition of α-GC, followed by the promotion of Treg expansion (Figs. 4, 6, and 7). Recent studies have identified NK-like-NKT1-related genes that can be distinguished from conventional NKT1-related genes in cytokine profile differencies^21,51^. Although subtypes of NKT1 cells have different roles in GVHD and the GVL effect remains unclear, excessive proinflammatory cytokine production by NK-like cells presumably deteriorates GVHD. Unfortunately, T-bet staining was unstable under severe cytopenia and could not be used for the definition of NKT1 subset in this study. To further delineate NKT subsets after BMT, it is needed to overcome the technical issue in flow cytometry. Also, the comprehensive gene analysis using a next-generation sequencer may help to widely explore the genes that can characterize the effect and function of NKT subsets.

In our analysis of T cell reconstitution after PTCy with lipo α-GC, lipo α-GC administration was involved in more enhanced early recovery of both CD4^+^ and CD8^+^ T cell subsets than PTCy alone (Fig. 7c). In particular, Treg recovery was remarkably promoted. Donor graft-derived Tregs in the early phase after PTCy with lipo α-GC treatment might preserve the effect on the late phase by subsequently inducing donor stem cell-derived Tregs. In terms of combination therapy, it is important to determine which immunosuppressive therapy is appropriate to combine with α-GC therapy. Previous studies have shown that donor-derived Tregs are indispensable for the suppression of GVHD by a single treatment with PTCy or α-GC after HSCT^26,49,52^. Our findings suggest that PTCy and α-GC function coordinately to increase donor-derived Treg numbers to induce tolerance. Although calcineurin inhibitors (CNIs) are widely used for GVHD prophylaxis, they have been shown to inhibit Tregs as well as alloreactive T cells^53^. To produce a synergistic effect with α-GC on Treg expansion, PTCy might be preferable to CNIs^52^.

In considering clinical applications, some differences in NKT cells between humans and mice must be acknowledged. In particular, the NKT cell population in mice is relatively large, and the number of human NKT cells is low. Therefore, the effect of NKT cells in human clinical transplantation may be relatively small compared to that in mouse.

To our knowledge, this is the first study to evaluate the efficacy of reduced-dose PTCy on the GVL effect in a murine PTCy-treated transplantation model. Our experiments clearly demonstrate that reducing the dose of PTCy can provide a promising strategy that enhances the GVL effect in this transplant setting. Our data may support clinical observations from a clinical trial which showed that reducing the dose of PTCy could result in prolonged survival among patients with severely high-risk diseases^54^. However, in the clinical trial, severe GVHD was also observed after reduced-dose PTCy, which counterbalanced the clinical merit of potentially enhancing the GVL effect. Our trials focusing on iNKT activation by multiple administrations of lipo α-GC as an immune adjuvant may establish a novel strategy to resolve the insufficient GVHD suppression mediated by reduced-dose PTCy and maintain a favorable balance between GVHD suppression and GVL enhancement. Considering the increasing clinical use of PTCy-based transplantation, the addition of a combination therapy should be considered to optimize this transplantation approach, promote restoration tolerance, and eradicate the disease in patients after HSCT.

## Methods

### Ethical statement

The study protocols were reviewed and approved by the Animal Care and Use Committee, Okayama University Advanced Science Research Center (protocol number: OKU-2017070 and OKU-2018489). All experiments were performed in accordance with the study protocols. The study was carried out in compliance with the ARRIVE guidelines.

### Mice

C57BL/6 J (H-2K^b^CD45.2^+^) mice and B6D2F1 (H-2 Kb^/d^CD45.2^+^) mice were purchased from Japan SLC (Shizuoka, Japan). C57BL/6-Ly5.1 (Ly5.1-B6, H-2K^b^CD45.1^+^) mice were purchased from RIKEN BRC (Tsukuba, Japan). In all experiments, 8- to 12-week-old female mice were used and maintained under specific pathogen-free conditions.

### Experimental transplantation

TCD-BM cells were obtained using CD90.2 microbeads and an Auto MACS system (Miltenyi Biotec, Bergisch Gladbach, Germany). B6D2F1 recipient mice received lethal 12 Gy TBI that was split into 2 doses at 6-h intervals to minimize gastrointestinal toxicities. On day 0, the recipient mice were injected intravenously with 5 × 10^6^ TCD-BM cells and 10 × 10^6^ splenocytes from B6 mice.

For assessment of the GVL effect, B6D2F1 recipient mice received sublethal 8 Gy TBI split into 2 doses. The recipient mice were transplanted as above, and 5 × 10^5^ luciferase-transduced P815 cells were injected intravenously.

### Treatment with PTCy and lipo α-GC

Cyclophosphamide was purchased from Sigma-Aldrich Japan (Tokyo, Japan). Ligands for iNKT cells, lipo α-GC (RGI-2001), were provided by REGiMMUNE Corp (Tokyo, Japan). Cyclophosphamide at a dose of 25 or 50 mg/kg was administered intraperitoneally into B6D2F1 recipient mice on day 3. Lipo α-GC at a dose of 1 μg/kg was intravenously administered on days 3, 5, and 7.

### Assessment of GVHD and the GVL effect

Survival after transplantation was monitored daily, and signs of clinical GVHD were assessed weekly by evaluating changes in weight, posture, activity, fur texture, and skin integrity, as previously described^55^. In a GVL model, death caused by leukemia was diagnosed by identifying hepatosplenomegaly, macroscopic tumor nodules in the liver or spleen, or hind-leg paralysis. Death caused by GVHD was identified by the absence of leukemia and the presence of clinical signs of GVHD. Tumor progression was also evaluated by bioluminescence imaging assay as shown in Supplemental Method.

### Flow cytometric analysis

Single-cell suspensions of splenocytes or mLN cells were incubated with anti-Fc receptor blocking antibodies and stained for surface markers with appropriate monoclonal antibodies (mAbs) for 30 min at 4 °C. To detect iNKT cells, cells were stained with R-PE-conjugated CD1d tetramers preloaded with α-GC, which were purchased from ProImmune (Oxford, England, UK). For intracellular staining, dead cells were first excluded with the Fixable Viability Dye (eBioscience), and cells were processed with the Foxp3 Transcription Factor Staining Buffer Set (eBioscience) according to the manufacturer’s protocol. Finally, the cells were stained for intracellular markers with appropriate mAbs. Samples were acquired with the MACS Quant Analyzer (Miltenyi Biotec), and data were analyzed with FlowJo software (Tree Star, Ashland, OR). The mAbs used in this study are shown in supplemental information.

### Statistical analysis

The Mann–Whitney U test and chi-square test were used to assess statistical significance between two groups, and one-way ANOVA was used to compare more than two groups. Tukey’s adjustment was used for multiple comparisons. The Kaplan–Meier method was used to obtain survival probabilities, and the log-rank test was applied to compare survival curves. Holm’s adjustment was used for multiple comparisons. *P* values < 0.05 were defined as statistically significant.

## Supplementary Information

Supplementary Information.

## Abbreviations

PTCy: Posttransplantation cyclophosphamide
HSCT: Hematopoietic stem cell transplantation
α-GC: α-Galactosylceramide
GVHD: Graft-versus-host disease
GVL: Graft-versus-leukemia
iNKT: Invariant natural killer T
APCs: Antigen-presenting cells
IFN: Interferon
TNF: Tumor necrosis factor
IL: Interleukin
PLZF: Promyelocytic leukemia zinc finger
Th1: Type 1 helper T
NKT1: Th1-biased NKT
Treg: Regulatory T
lipo α-GC: Liposome formulation containing α-GC
TCD: T cell-depleted
BM: Bone marrow
B6: C57BL/6 J
TBI: Total body irradiation
IVIS: In vivo imaging system
DCs: Dendritic cells
HSC: Host stem cell
CNIs: Calcineurin inhibitors
