# Supplementary material for: Reduced dose of PTCy followed by adjuvant α-galactosylceramide enhances GVL effect without sacrificing GVHD suppression
Source: Sci Rep. 2021 Jun 23;11:13125. doi: 10.1038/s41598-021-92526-z (PMC8222309; doi:10.1038/s41598-021-92526-z)
Supplement: Supplementary file 1 — Supplementary Information. [file 41598_2021_92526_MOESM1_ESM.pdf]

Supplementary Materials for

Reduced dose of PTCy followed by adjuvant α-galactosylceramide enhances GVL effect without sacrificing GVHD suppression

Authors:

Makoto Nakamura[1], Yusuke Meguri[1], Shuntaro Ikegawa[1], Takumi Kondo[1], Yuichi Sumii[1], Takuya Fukumi[1], Miki Iwamoto[1], Yasuhisa Sando[1], Hiroyuki Sugiura[1], Noboru Asada[2], Daisuke Ennishi[2,3], Shuta Tomida[3], Emi Fukuda-Kawaguchi[4,5], Yasuyuki Ishii[4,6], Yoshinobu Maeda[1,2], and Ken-ichi Matsuoka[1,2]

Affiliations:

[1] Department of Hematology and Oncology, Okayama University Graduate School of Medicine, Dentistry and Pharmaceutical Sciences, Okayama, Japan

[2] Department of Hematology and Oncology, Okayama University Hospital, Okayama, Japan

[3] Center for Comprehensive Genomic Medicine, Okayama University Hospital, Okayama, Japan

[4] REGiMMUNE Corporation, Tokyo, Japan

[5] Department of Urology, Tokyo Women's Medical University, Tokyo, Japan

[6] Department of Immunological Diagnosis, Juntendo University Graduate School of Medicine, Tokyo, Japan

This file includes: Supplemental method and Supplemental Figure1-4

Corresponding Author:

Ken-ichi Matsuoka M.D., Ph.D

Department of Hematology and Oncology, Okayama University Graduate School of Medicine, Dentistry, and Pharmaceutical Sciences, Okayama, Japan

Phone: +81-86-235-7227

Fax: +81-86-232-8226

E-mail: k-matsu@md.okayama-u.ac.jp

Supplemental Method

Antibodies for flow cytometry

The following mAbs used in this study were purchased from BD Biosciences (San Jose, CA, USA), BioLegend (San Diego, CA, USA), eBioscience (San Diego, CA, USA) or R&D Systems (Minneapolis, MN, USA): eFluor450-conjugated anti-B220 (RA3-6B2), eFluor450-conjugated anti-CD4 (GK1.5), PB-conjugated anti-TCRβ (H57-597), FITC-conjugated anti-CD4 (RM4-5), FITC-conjugated anti-H-2Kd (SF1-1.1.1), PE-conjugated anti-H-2Kd (SF1-1.1.1), PE-conjugated anti-CD45.1 (A20), PE-Cy7-conjugated anti-CD25 (PC61.5), PE-Cy7-conjugated anti-CD45.1 (A20), APC-conjugated anti-CD11c (N418), APC-conjugated anti-CD19 (MB19-1), APC-eFluor780-conjugated anti-CD8a (53-6.7), APC-eFluor780-conjugated anti-CD11b (M1/70), APC-eFluor780-conjugated anti-CD19 (1D3), APC-Cy7-conjugated anti-CD3 (17A2), PE-Cy7-conjugated anti-PLZF (9E12), APC-conjugated anti-Foxp3 (FJK-16s), and Alexa Fluor647-conjugated anti-RORγt (Q31-378).

Evaluation of the GVL effect by bioluminescence imaging.

In a GVL model, 3 mg of D-Luciferin (OZ Biosciences USA, San Diego, CA, USA) were injected intraperitoneally into each mouse, and the tumor burden was assessed by bioluminescence imaging (BLI) on days 17, 24, 31, 38, and 45. BLI was performed with an IVIS (IVIS Lumina, Xenogen, Alameda, CA, USA). Images were analyzed with Living Image Software 3.2 (Xenogen).

Supplemental Fig. 1

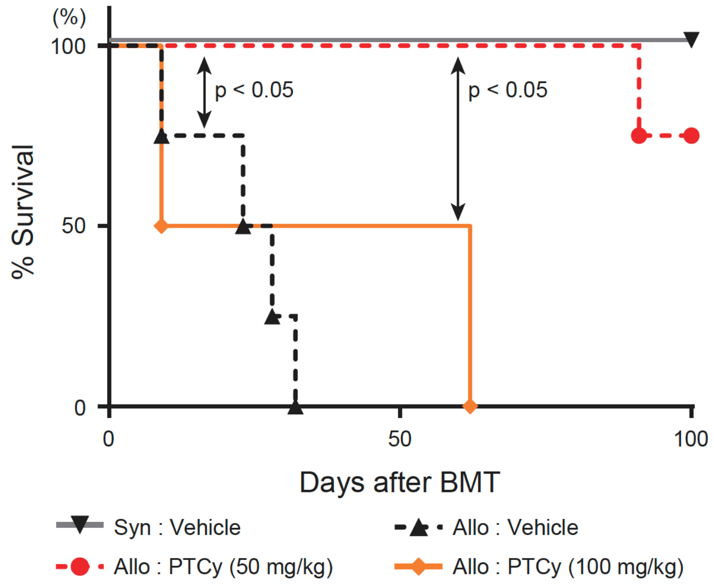

5 × 10^6 TCD-BM cells and 10 × 10^6 splenocytes from B6 donor mice were administered to lethally irradiated (12 Gy TBI) B6D2F1 recipient mice. Two different doses of cyclophosphamide (50 mg/kg or 100 mg/kg) or a control vehicle was administered on day 3 after BMT. PTCy at a dose of 50 mg/kg significantly protected recipients from acute GVHD, while a dose of 100 mg/kg failed to do so. Overall survival rates were compared among Syn: Vehicle (n = 4), Allo: Vehicle (n = 4), Allo: 50 mg/kg PTCy (n = 4) or Allo: 100 mg/kg PTCy (n = 4). Data is obtained from one experiment. P values by log-rank test and the Holm's adjustment for multiple comparison.

Supplemental Fig. 2

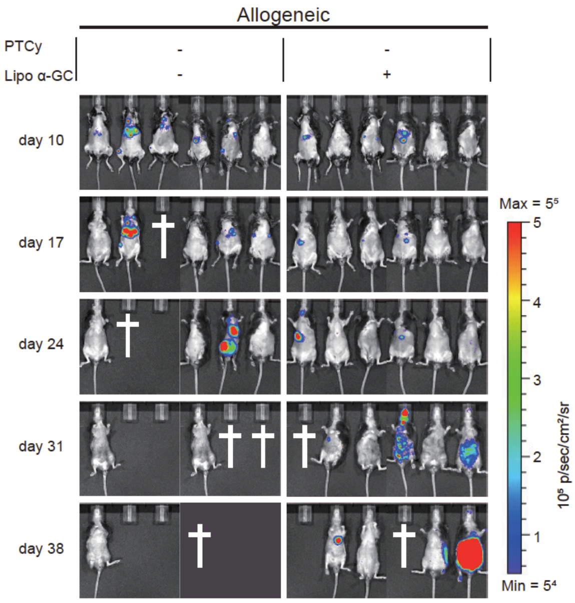

IVIS study of the allogeneic cohorts without PTCy.

The results from groups with or without lipo α-GC (n = 6 in each group) at serial time points are shown.

Supplemental Fig. 3

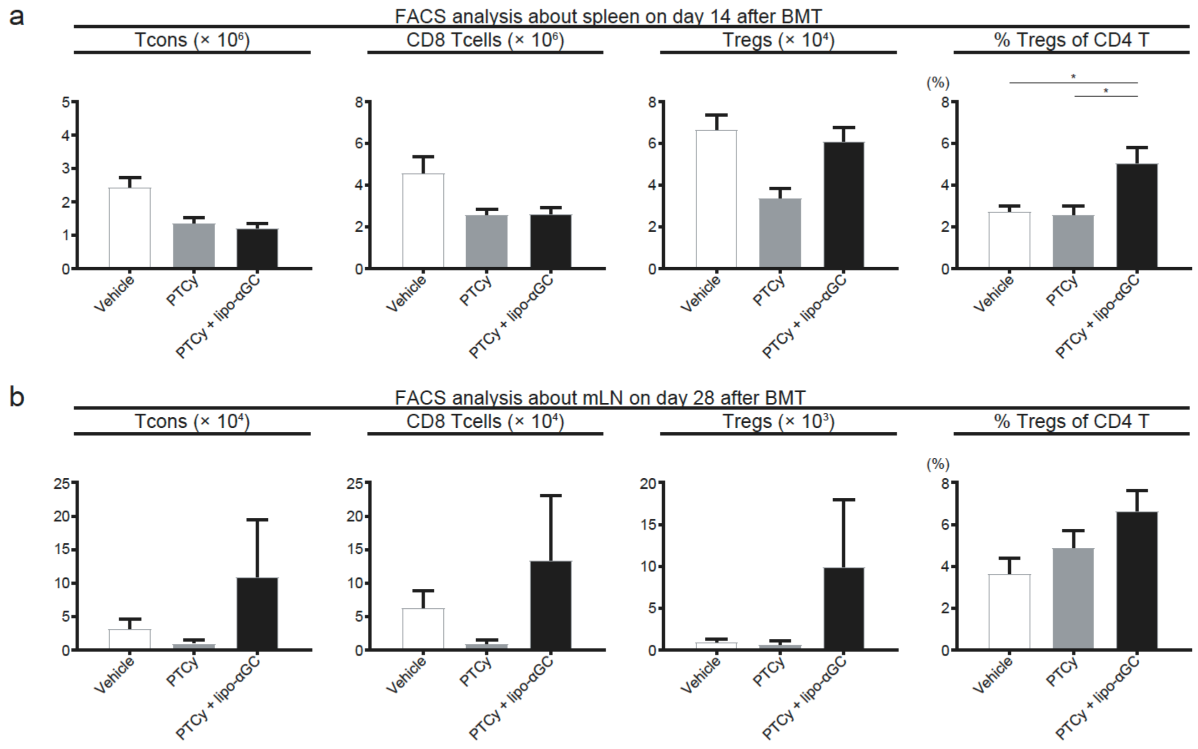

(a) FACS analysis of splenocytes on day 14 after BMT revealed the absolute number of Tcons, CD8 T cells and Tregs and percentages of Tregs. (b) FACS analysis of mLN cells on day 28 after BMT revealed the absolute number of Tcons, CD8 T cells and Tregs and percentages of Tregs. P values by one-way ANOVA and the Tukey's adjustment for multiple comparison. * $P < .05$.

Supplemental Fig. 4

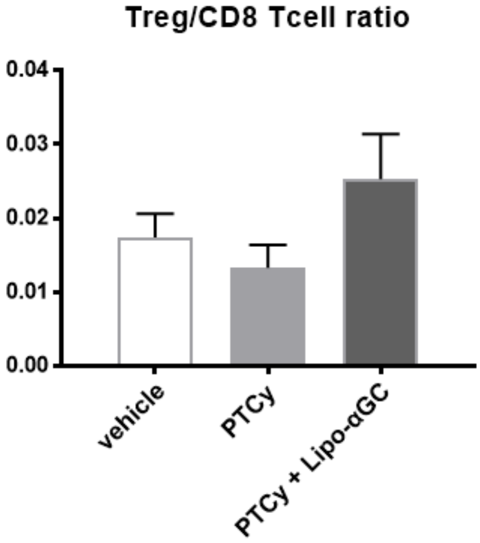

Flow cytometric analysis of mLN cells on day 14 after BMT revealed the Treg to $CD8^+$ T cells ratio in each group.
